# Y-chromosomal diversity of the Valachs from the Czech Republic: model for isolated population in Central Europe

**DOI:** 10.3325/cmj.2011.52.358

**Published:** 2011-06

**Authors:** Edvard Ehler, Daniel Vaněk, Vlastimil Stenzl, Václav Vančata

**Affiliations:** 1 Department of Anthropology and Human Genetics, Faculty of Science, Charles University in Prague, Prague, Czech Republic; 2 Department of Biology and Environmental Studies, Faculty of Education, Charles University in Prague, Prague, Czech Republic; 3 Forensic DNA Service, Prague, Czech Republic; 4 2nd Faculty of Medicine, Charles University in Prague, Prague, Czech Republic; 5 Department of Forensic Genetics, Institute of Criminalistics, Prague, Czech Republic; 6 Institute of Anthropology, Faculty of Science, Masaryk University, Brno, Czech Republic

## Abstract

**Aim:**

To evaluate Y-chromosomal diversity of the Moravian Valachs of the Czech Republic and compare them with a Czech population sample and other samples from Central and South-Eastern Europe, and to evaluate the effects of genetic isolation and sampling.

**Methods:**

The first sample set of the Valachs consisted of 94 unrelated male donors from the Valach region in northeastern Czech Republic border-area. The second sample set of the Valachs consisted of 79 men who originated from 7 paternal lineages defined by surname. No close relatives were sampled. The third sample set consisted of 273 unrelated men from the whole of the Czech Republic and was used for comparison, as well as published data for other 27 populations. The total number of samples was 3244. Y-short tandem repeat (STR) markers were typed by standard methods using PowerPlex® Y System (Promega) and Yfiler® Amplification Kit (Applied Biosystems) kits. Y-chromosomal haplogroups were estimated from the haplotype information. Haplotype diversity and other intra- and inter-population statistics were computed.

**Results:**

The Moravian Valachs showed a lower genetic variability of Y-STR markers than other Central European populations, resembling more to the isolated Balkan populations (Aromuns, Csango, Bulgarian, and Macedonian Roma) than the surrounding populations (Czechs, Slovaks, Poles, Saxons). We illustrated the effect of sampling on Valach paternal lineages, which includes reduction of discrimination capacity and variability inside Y-chromosomal haplogroups. Valach modal haplotype belongs to R1a haplogroup and it was not detected in the Czech population.

**Conclusion:**

The Moravian Valachs display strong substructure and isolation in their Y chromosomal markers. They represent a unique Central European population model for population genetics.

Y-chromosomal variation of Central European populations and the possible appearance of genetic isolates in these populations are of increasing interest to forensic and human population geneticists.

Y-chromosomal data for the population of the Czech Republic is still fractional. Kráčmarová et al published a short report on paleolithic and neolithic Y chromosomal haplogroups in the Czech population (1) and Luca et al performed a refined study of the same data (2). Zastera et al published a major study on Czech Y-chromosomal data (3). Other authors have also reported on Czech Y-chromosomal variation, usually with other population data from Europe (4-7). A recent study compared Czechs with other West Slavic populations (8). In this range of reports regarding, genetic variation of possible or confirmed genetic isolates within Central European populations is virtually absent. Here we present the intra-population diversity of such an isolated population, the Moravian Valachs.

So far, a limited number of studies that illustrate the variety of Y-chromosomal polymorphisms in the countries and populations supposedly connected or similar to the Moravian Valachs – the supposed isolate – have been published. Rebala et al (9) focused on the Slavic population from Eastern and Central Europe. As historical sources suggest, immigration from Slavic populations was one of the major sources for the emergence of the Valach population of the Czech Republic, therefore the study of Rebala et al (9) is certainly of great interest to us, as well as other studies on southern European Slavic populations (10). Bosch et al (11) analyzed paternal (and maternal) lineages of the Aromuns and other surrounding Balkan populations, thus offering excellent material for their comparison with the Valachs. They clearly documented the differences between Aromuns (ie, isolated populations) and the major populations that surround them, not only in haplogroup and haplotype lineages, but also in intra-population genetic variability.

The Valachs (or Wallachs/Vlachs as they are sometimes called) are one of the most distinct ethnographic and cultural subpopulations of Central Europe. Today, they can be found not only in the Czech Republic – in its eastern border mountain ranges and highlands (Beskydy in Moravia) – but also in south-southeast Poland and several parts of Slovakia (far western, northern, and central region). Originally, this group spread from the Maramures region of Romania, roughly following the Carpathian Mountain range. The arrival of the Valachs to the area of today’s Czech Republic took place at the very end of the 15th or beginning of the 16th century (12). The migration was not spontaneous, but rather encouraged and subsidized by the local nobility, and it lasted at least until the end of the 18th century, with immigrants supposedly coming not only from Romania, but also from Ukraine, Poland, and Slovakia (13).

Until the beginning of the 20th century, the Moravian Valachs’ way of life was similar to other Romanian ethnic groups in the Balkans, especially the Aromuns (seasonal mountain sheep herding, production of cheese, wool, and leather products). An admixture of the newly-arrived Valachs with autochthonous (Slavic and German) Moravian population also began soon after the arrival of the first immigrants – so we can assume a steady genetic and cultural flow between these two populations. Nonetheless, the core of the Valach settlement was located in a previously uninhabited high altitude region, neighboring with the indigenous population from lowlands. The result of the admixture process was a complete merging of both populations, and the disappearance of any distinction between “new” Valachs and indigenous Moravians during the 18th century, and the creation of one ethnogeographic region with all its properties and people – the Moravian Valachs.

Demographic data (13,14) show only a small increase in the Valach population during the 17-18th century. In combination with population depression during and after the Thirty Years' War (1618-1648), the conditions in the Valach population favored inbreeding, an effect reinforced by isolation-by-distance from the surrounding populations.

To investigate how severe this isolation effect was on Y chromosomal polymorphisms in the Moravian Valachs and whether it is still detectable in modern Valach population is the main aim of our study. Another topic of interest was how the intra-population variability and the sampling bias can affect forensic and population analyses performed on these data.

## Material and methods

Hundred and seventy-three DNA samples of male Valachs from the Czech Republic were analyzed. These samples were divided into two groups because of the important differences in sampling procedure and are consistently referred to separately throughout this article.

The first group consisted of 94 samples of unrelated donors (code: VALACH, Moravian Valachs). All donors identified themselves as belonging to the Valach ethnic group in a short interview that was held immediately before DNA sampling in the form of mouth swabs. Only donors whose paternal lineage was present in the region of the Valach country for at 3 generations were included into the study. Informed consent was provided by the donors and no other data (including name, address, etc.) were gathered. The data were rendered fully anonymous.

The second Valach sample set consisted of 79 samples (code: VLIN, Moravian Valachs lineages). The sampling process in this case differed significantly from the VALACH sample set. VLIN sample set came from 7 Valach paternal lineages. These were defined primarily by surname, as well as by geographic localization in the Valach region and self-identification of the donors. Although the samples came from broad families, no first, second, third, and fourth degree relatives were included in the study, virtually making this Valach sample set composed of unrelated, non-randomly selected Valachs, carrying 7 different surnames.

The control sample set consisted of 273 unrelated male donors from the whole Czech Republic. Donors did not identify themselves as having the Valach origin, however, there was no other information gathered about their ethnicity or origin. Data are available on request and will be submitted to the Y-chromosome haplotype reference database (*http://www.yhrd.org/)* database.

We gathered published samples for Y-short tandem repeat (STR) loci from other populations, concentrating on Eastern European and Balkan populations. Our total set, Moravian Valachs included, consisted of 30 populations encompassing 3244 individuals (Table 1). Due to the limitation of the published data, only minimal haplotype loci (DYS19, DYS389I, DYS389II, DYS390, DYS391, DYS392, DYS393, and DYS385a/b) were used for the analysis of intra-population statistics computing and the comparison between populations. For detailed analysis, 12 loci haplotypes were utilized, which also included all extended haplotype loci (minimal haplotype loci + DYS437, DYS438, DYS439).

**Table 1:** Population set used in this study

**Population**	**Code**	**Data source**	**Number of samples**
Albanians (Tirana)	ALB	(11)	34
Aromuns (Romania)	ARO	(11)	48
Aromuns (Andon Poci, Albania)	AAA	(11)	19
Aromuns (Dukasi, Albania)	AAD	(11)	39
Aromuns (Krusevo, Macedonia)	AMK	(11)	43
Aromuns (Stip, Macedonia)	AMS	(11)	58
Bulgarian Gypsies	BUG	(35)	81
Bulgarian Turks	BUT	(35)	61
Bulgars	BUL	(35)	122
Croatians (Rijeka area)	CRO	(36)	101
Csango (Lunca de Sus)	CSA	(37)	84
**Czechs (Czech Republic)**	**CZE**	**this study**	**273**
Greeks (Thrace)	GRE	(11)	39
Hungarians (Budapest)	HUN	(38)	116
Albanians (Kosovo)	ALK	(29)	117
Luzice Sorbs	SORB	(32)	29
Macedonians	MACE	(39)	84
Macedonian Romani	RMA	(39)	68
Macedonians (Skopje)	MAC	(11)	51
**Moravian Valachs**	**VALACH**	**this study**	**94**
**Moravian Valachs (lineages)**	**VLIN**	**this study**	**79**
Poles (Central Poland)	POLC	(40)	254
Poles (SE. Poland)	POSE	YA003352*	161
Romanians (Constanta)	ROMC	(11)	31
Romanians (Ploesti)	ROMP	(11)	36
Russians (European part)	RUS	(41)	541
Saxons (Dresden)	SAX	(32)	89
Serbia and Monte Negro	SEMN	(42)	237
Slovaks (Bratislava)	SLO	(9)	164
Szekely (Miercurea Ciuc)	SZE	(37)	91
**Total**			**3244**

*POSE population comes from *http://www.yhrd.org*/ database, accession number provided.

The Y-chromosomal STRs of VALACH samples were assessed using PowerPlex® Y System (Promega, Madison, WI, USA). It contained 12 microsatellite polymorphic sites (DYS19, DYS389I, DYS389II, DYS390, DYS391, DYS392, DYS393, DYS385a/b, DYS437, DYS438, and DYS439), including all recommended minimal haplotype loci (minHT), and all extended haplotype loci (extHT; SWGDAM recommended loci).

CZE samples and VLIN samples were typed using AmpFiSTR Yfiler® PCR Amplification Kit (Applied Biosystems, Carslbad, CA, USA), that included all of the above mentioned loci plus DYS456, DYS458, DYS635, Y GATA H4, DYS448. All 17 markers were used only in evaluating discrimination capacity of Y-STR haplotypes in CZE and VLIN sample sets.

Using the Y-STR information, we estimated also the Y chromosomal haplogroups in our samples by the free internet software tool ‘Haplogroup Predictor’ by Whit Athey (*http://www.hprg.com/hapest5/*) (15,16). We were aware of the issues present in estimating Y-chromosomal haplogroups from Y-STR frequencies (17), thus for the subsequent analyses (median networks) we used only the samples with Hg estimate probability higher than 90%.

Genetic intra-population indices were computed in Arlequin v3.1 software (18). For construction of median networks, we used Neworks 4.60 (available at *http://www.fluxus-engineering.com*) (19,20). We used 12 Y-STR loci (DYS19, DYS389I, DYS389II, DYS390, DYS391, DYS392, DYS393, DYS385a/b, DYS437, DYS438, and DYS439) for the network analysis. A reduced median algorithm (r = 2) was followed by a median joining procedure (epsilon = 0) to reduce the reticulation of the networks (21). The results from the median joining procedure were post-processed by maximum parsimony calculation to further simplify the final network.

The multidimensional scaling analysis was performed in Statistica 9.0 software (StatSoft Inc., Tulsa, OK, USA).

## Results

The Moravian Valachs of the Czech Republic showed remarkably low values of intra-population genetic diversity. This low differentiation, as compared with other populations in our study, is shown by the haplotype (gene) diversity values ± standard deviation (0.9792 ± 0.0075), the average gene diversity per locus (0.476607 ± 0.268081), and the mean number of pairwise differences (3.812857 ± 1.936064) (Table 2). This is especially true if we compare the Valachs to population samples from adjacent populations, ie, Czechs, Slovaks, Saxons, and both Polish samples. In this comparison, the Valachs’ Y-chromosomal variability was lower, and their haplotypes were more similar to each other. Our second Valach data set of Valach lineages (VLIN) showed even more extreme values of haplotype diversity – the 5th lowest value from our population set (0.9335 ± 00192) – which can be expected given that they come from paternal lineages. The average diversity per locus of the VLIN data set (0.573799 ± 0.315593) and the mean number of pairwise differences (4.590393 ± 2.278308) were still lower than the cross-population average, but not as extreme as their haplotype diversity.

**Table 2:** Intrapopulation genetic diversity parameters of our population sample set

Population	Code	Gene (haplotype) diversity*			Average gene diversity over loci*		Mean number of pairwise differences*	
			SD^†^	Rank^‡^		SD^†^		SD^†^
Albanians (Kosovo)	ALK	0.9621	0.0079	7	0.546309	0.301059	4.370469	2.175093
Albanians (Tirana)	ALB	0.9911	0.0093	14	0.608734	0.338317	4.869875	2.434188
Aromuns (Andon Poci, Albania)	AAA	0.7076	0.0690	1	0.462719	0.273324	3.701754	1.956638
Aromuns (Dukasi, Albania)	AAD	0.7976	0.0615	2	0.498988	0.283267	3.991903	2.039691
Aromuns (Krusevo, Macedonia)	AMK	0.9911	0.0077	15	0.612957	0.338176	4.903654	2.436244
Aromuns (Romania)	ARO	0.9645	0.0141	8	0.644393	0.352587	5.155142	2.541297
Aromuns (Stip, Macedonia)	AMS	0.9062	0.0204	4	0.566773	0.313685	4.53418	2.262519
Bulgarian Gypsies	BUG	0.9556	0.0149	6	0.53561	0.297067	4.284877	2.144699
Bulgarian Turks	BUT	0.9978	0.0035	28	0.66127	0.359114	5.290164	2.590619
Bulgars	BUL	0.9977	0.0015	27	0.617328	0.33504	4.938626	2.420761
Croatians (Rijeka area)	CRO	0.9949	0.0030	21	0.618688	0.336276	4.949505	2.42891
Csango (Lunca de Sus)	CSA	0.9791	0.0057	9	0.626757	0.340851	5.014056	2.46101
**Czechs (Czech Republic)**	**CZE**	**0.9964**	**0.0010**	**26**	**0.625512**	**0.337410**	**5.004094**	**2.439948**
Greeks (Thrace)	GRE	0.9906	0.0100	13	0.618084	0.341532	4.944669	2.459228
Hungarians (Budapest)	HUN	0.9987	0.0013	30	0.659389	0.355352	5.275112	2.567318
Luzice Sorbs	SORB	0.9852	0.0136	11	0.5508	0.311564	4.406404	2.239319
Macedonian Romani	RMA	0.9008	0.0242	3	0.477996	0.269877	3.823968	1.947508
Macedonians	MACE	0.9885	0.0058	12	0.583333	0.319942	4.666667	2.310046
Macedonians (Skopje)	MAC	0.9929	0.0058	17	0.647843	0.353808	5.182745	2.550724
**Moravian Valachs**	**VALACH**	**0.9792**	**0.0075**	**10**	**0.476607**	**0.268081**	**3.812857**	**1.936064**
**Moravian Valachs (lineages)**	**VLIN**	**0.9335**	**0.0192**	**5**	**0.573799**	**0.315593**	**4.590393**	**2.278308**
Poles (Central Poland)	POLC	0.9957	0.0012	23	0.570041	0.311005	4.560331	2.248893
Poles (SE. Poland)	POSE	0.9929	0.0024	18	0.547438	0.300886	4.379503	2.174803
Romanians (Constanta)	ROMC	0.9978	0.0089	29	0.636828	0.353164	5.094624	2.539503
Romanians (Ploesti)	ROMP	0.9952	0.0078	22	0.604563	0.335687	4.836508	2.416084
Russians (European part)	RUS	0.9943	0.0009	20	0.60807	0.328484	4.864558	2.376197
Saxons (Dresden)	SAX	0.9959	0.0030	25	0.632629	0.343441	5.061032	2.480027
Serbia and Monte Negro	SEMN	0.9917	0.0024	16	0.571837	0.311953	4.574698	2.255625
Slovaks (Bratislava)	SLO	0.9932	0.0021	19	0.590762	0.32161	4.726096	2.324641
Szekely (Miercurea Ciuc)	SZE	0.9958	0.0029	24	0.679151	0.365707	5.433211	2.640936

*The descriptive statistics were computed from the Y-short tandem repeat haplotype frequencies based on minimal haplotype loci (DYS19, DYS389I, DYS389II, DYS390, DYS391, DYS392, DYS393, and DYS385a/b).

^†^SD – standard deviation.

^‡^The ranking sequence according to the gene diversity values (from the lowest to the highest).

To assess the power of discrimination of the used Y-STR markers, we computed the discrimination capacity for both of the Valach sample sets (VALACH, VLIN) and Czech sample set (CZE) for 9, 12, and 17 Y-STR marker haplotypes (Table 3). Discrimination values of Y-STR haplotypes were markedly low in VLIN, which is due to the effect of the sampling procedure.

**Table 3:** Comparison of discrimination capacity of 9, 12, and 17 Y-short tandem repeat (STR) loci haplotypes in two Moravian Valach sample sets and Czech population sample set

**Code**	**n**	9 Y-STR (minHT) loci*			**12 Y-STR loci (PowerPlex Y)^†^**			**17 Y-STR loci (Yfiler)^‡^**		
		No. of haplotypes	discrimination capacity (%)	gene diversity	No. of haplotypes	discrimination capacity (%)	gene diversity	No. of haplotypes	discrimination capacity (%)	gene diversity
**VALACH**	94	66	70.21	0.9792	72	76.60	0.9840	N/A	N/A	N/A
**VLIN**	79	35	44.30	0.9335	39	49.37	0.9507	49	62.03	0.9786
**CZE**	273	214	78.39	0.9964	247	90.48	0.9987	266	97.44	0.9998

*DYS19, DYS389I, DYS389II, DYS390, DYS391, DYS392, DYS393, DYS385a/b.

^†^* + DYS437, DYS438, DYS439.

^‡^* + † + DYS456, DYS458, DYS635, Y GATA H4, DYS448.

**Table 4.** Genetic distances (F_ST_ – below diagonal; R_ST_ – above diagonal*) between Valach populations (VALACH, VLIN) and the geographically surrounding populations

**Figure 1:**
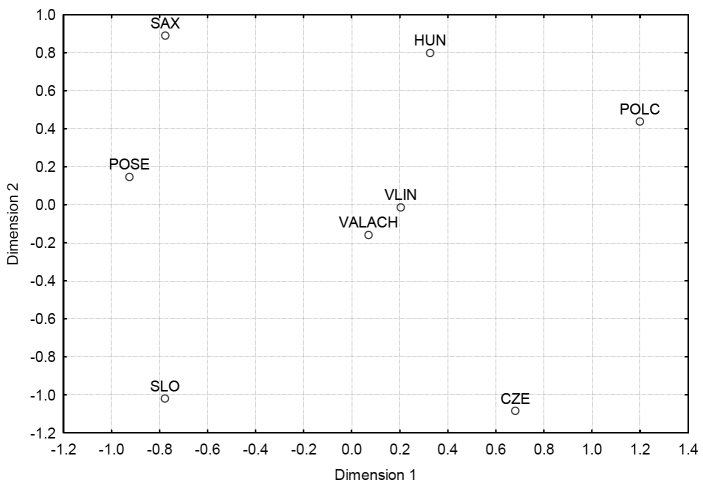
Plot of the first two dimensions of the multidimensional scaling analysis (stress value = 0.163) from the F_ST_ genetic distance matrix. VALACH – Moravian Valachs, VLIN – Moravian Valachs (lineages), CZE – Czechs, SLO – Slovaks, SAX – Saxons, HUN – Hungarians, POLC – Poles from Central Poland, POSE – Poles from Southeast Poland.

The values of intra-population statistics showed a decrease in Y-chromosomal diversity as the result of isolation. Further evidence of the isolation are the pairwise genetic distances between the Valachs (VALACH, VLIN) and other surrounding populations from our sample set: Czechs (CZE), Slovaks (SLO), Saxons (SAX), Hungarians (HUN), and Poles from Central and Southeast Poland (POLC, POSE). The pairwise F_ST_ distances between the Valachs and other populations were all significant at 0.10% level (10 000 permutations). The R_ST_ distances displayed a more random pattern, which could have been caused by small allele length differences between the populations. We computed the complete F_ST_ and R_ST_ genetic distances between the selected populations (Table 4). The F_ST_ genetic distances were visualized by multidimensional scaling analysis (MDS). Its results for first and second MDS dimensions are plotted in Figure 1. The Valachs showed marked genetic isolation from the neighboring populations.

**Figure 2:**
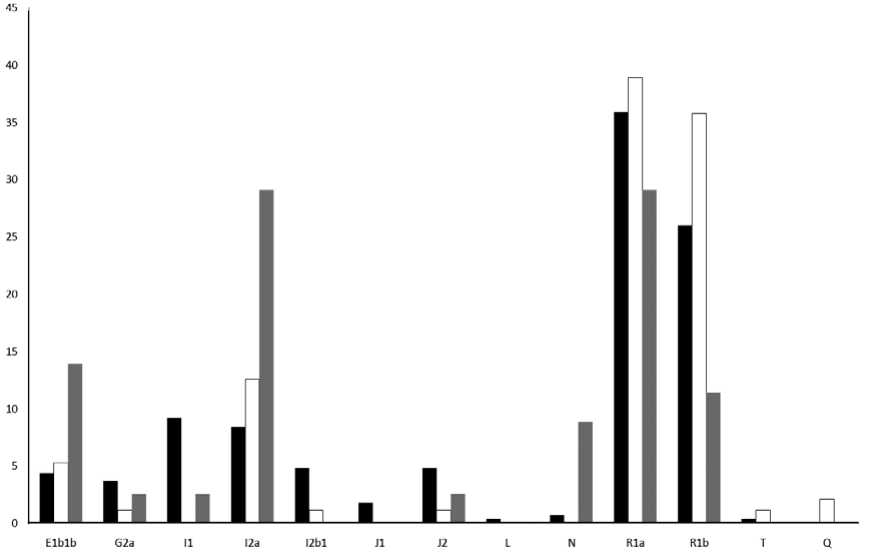
Distribution of Y chromosomal haplogroups in Moravian Valach (closed bars), Moravian Valach (lineages) (gray bars), and Czech (open bars) populations.

Distribution of Y chromosomal haplogroups in the VALACH, VLIN, and CZE populations was not uniform (Figure 2). While our Czech population sample well reflected the Central European Y haplogroup pool, the Valach sample set showed some deviation from the expected frequencies of the Y-haplogroups. This was especially noticeable in the VLIN sample set, with the overrepresentation of haplogroup I2a and N, each of them being a dominant haplogroup in one of the 7 paternal lineages sampled.

**Figure 3:**
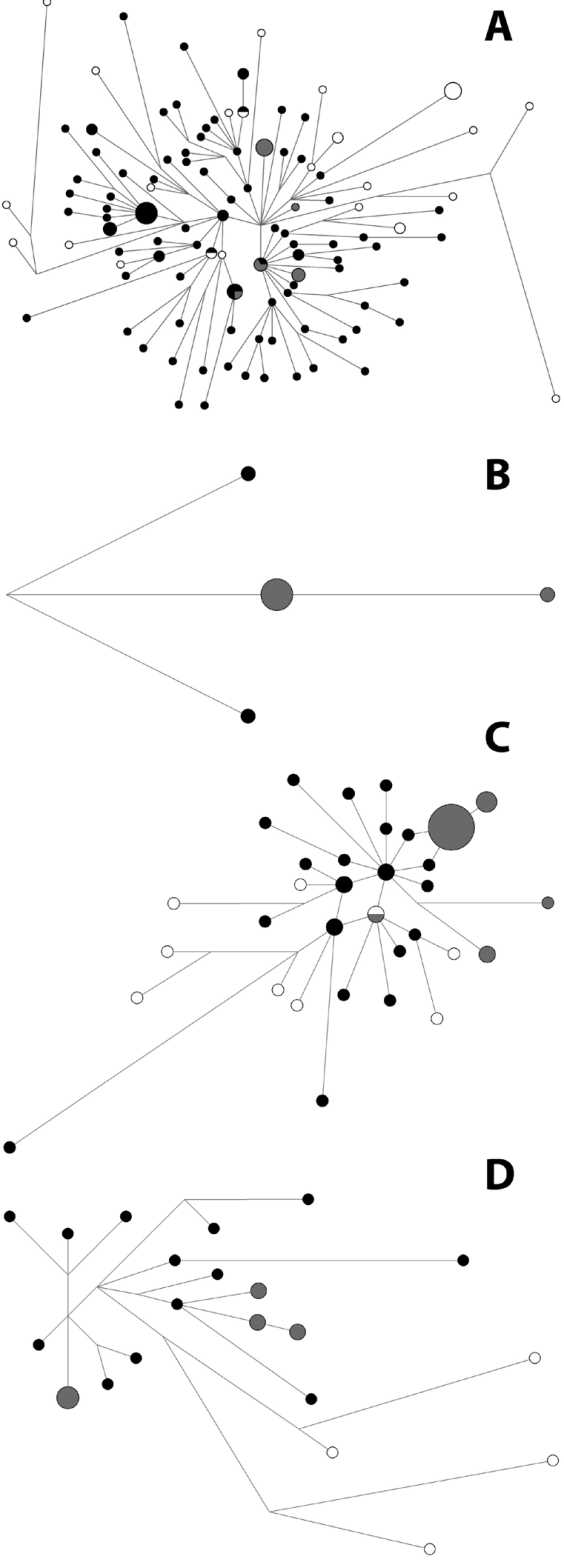
Median networks diagrams of internal Y chromosomal haplogroups variability for Czech (black), Moravian Valach (white), and Moravian Valach (lineages) (gray) sample sets. (**A**) Haplogroup R1a; (**B**) Haplogroup N; (**C**) Haplogroup I2a; (**D**) Haplogroup E1b1b.

Variation within selected haplogroups is displayed in Figure 3. Haplogroups R1a, N, I2a, and E1b1 were chosen because of their major representation in different paternal lineages of VLIN sample set. Marked isolation of Valach haplotypes within the haplogroups can be seen in R1a, I2a and E1b1b networks. This reflects the substructure of the examined populations. The effect of sampling can be seen when we compare the distribution of the VLIN and VALACH haplotypes (Figure 3). Closely related paternal lineages of the VLIN sample set demonstrate as clustered, low diversity, branches within the networks. Unrelated VALACH samples, while they still form almost exclusive Valach branches of the network, are separated by more mutation steps and exhibit higher diversity. VLIN in N and I2a come each from a different surname-defined paternal lineage. VLIN samples belonging to E1b1b originated in two paternal lineages. The correspondent clusters are clearly seen in Figure 3D. VLIN samples in R1a are from 3 paternal lineages with an intermingled substructure.

R1a is the most prevalent and the most diverse haplogroup in the Valach and Czech population. Therefore, we selected this haplogroup for further analysis and identification of its modal haplotype.

### Valach-specific modal haplotype

The Y-chromosome haplotype that is common in the Euro-Atlantic populations is called “Atlantic Modal Haplotype” (22). We propose that the common haplotype found in the population sample of 94 unrelated Moravian Valachs is called “Valach Modal Haplotype” (VMH). This modal haplotype belongs to the R1a haplogroup and is defined by 8 Y-STR polymorphisms (DYS390, DYS391, DYS392, DYS393, DYS385a, DYS385b, DYS437, DYS439) (Table 5). The comparison of the VMH with the control population samples CZE (n = 273) used in this study or the other published Czech population sample (3) (n = 1148) resulted in no matches. Further comparison with the European metapopopulation of Y-chromosome haplotype reference database (YHRD) (23,24) release 35 (built on December 30, 2010, 12:56 GMT, consisting of 91 601 haplotypes within 710 populations) resulted in 2 exact matches and a set of matches with neighbor haplotypes (difference of 1 repeat in 1 DYS included in the VMH) (Table 6).

**Table 5:** Valach Modal Haplotype within the R1a haplogroup in population sample of 94 unrelated Moravian Valachs (VALACH).

Haplotype	n	DYS19	DYS389I	DYS389II	DYS390	DYS391	DYS392	DYS393	DYS385a	DYS385b	DYS437	DYS439
VALACH8	4	14	13	29	24	10	11	13	11	14	15	12
VALACH15	1	14	13	30	24	10	11	13	11	14	15	12
VALACH19	5	14	13	31	24	10	11	13	11	14	15	12
VALACH38	2	15	13	29	24	10	11	13	11	14	15	12
VALACH52	2	16	13	30	24	10	11	13	11	14	15	12
VALACH55	1	16	13	31	24	10	11	13	11	14	15	12
VALACH72	1	18	13	30	24	10	11	13	11	14	15	12
**VALACH MODAL HAPLOTYPE**					**24**	**10**	**11**	**13**	**11**	**14**	**15**	**12**

**Table 6:** Comparison of Valach Modal Haplotype* with the European metapopulation

Locus difference^†^	Population^‡^, number of matches, (YHRD code)
**exact match**	Slovakia, 1x, (YA003557); Argentina, 1x, (YA003561)
DYS392 = 12	Bihor, Romania, 2x, (YA003605); Majorca, Spain, 1x, (YA003489)
DYS392 = 10	Taktakoz Romani, Hungary, 1x, (YA003659)
DYS391 = 11	Novi Sad, Serbia, 3x, (YA003632)
DYS437 = 14	Ivanava, Belorus, 3x, (YA003507); Santa Ninfa, Italy, 2x, (YA003110); Novi Sad, Serbia, 1x, (YA003632); Rjasan, Russia, 1x, (YA003178); Klimaviči, Belarus, 1x, (YA003508); Stuttgart, Germany, 1x, (YA003031); Cologne, Germany, 1x, (YA002963)
DYS390 = 25	Sverdlovsk, Russia, 1x, (YA003678)
DYS393 = 12	Eastern Slovakia, 1x, (YA003546)
DYS439 = 13	Eastern Slovakia, 1x, (YA003546)

*Valach Modal Haplotype: 24-10-11-13-11-14-15-12; DYS390-391-392-393-385a-385b-437-439.

^†^Exact matches and 1 allele difference matches are shown.

^‡^European metapopulation from YHRD database, release 35 (*http://www.yhrd.org/*).

## Discussion

We found the traces of isolation and substructure in the Moravian Valachs’ Y-chromosomal genetic variation. Studies with well defined Y-chromosomal data for Central Europe are scarce. Previously mentioned studies of Czech population Y-STR variability reported no inner differentiation of the population (1,3). A substructure of Y-chromosomal lineages was reported in the Brabant region of Belgium and the Netherlands (25). Also, a strong Y-chromosomal breakpoint in Romanian population, based on ethnic origin, was demonstrated (26). Petrejčíková et al (27) have analyzed men from Eastern Slovakia and found non-significant separation from the surrounding Slavic populations. In Belarus population, a limited population substructure was observed (28), although only detectable when using 7-12 Y-STR haplotypes. With 17 Y-STR haplotypes (Yfiler loci), the substructure was no longer detectable. The population of Moravian Valachs analyzed in our study displayed signs of isolation and substructure, which are noticeable in 9, 12, and 17 Y-STR haplotypes. The isolation of the Valach population, low effective population size and, thus, the faster operation of genetic drift are expressed in low haplotype diversity in the Valachs. These effects of isolation are also evident in the average diversity values over loci and the mean number of pairwise differences. Regarding these parameters, the Moravian Valachs more resemble rather isolated Balkan populations (Aromuns, Csango, Bulgarian Roma, and Macedonian Roma) (11,29), population from eastern Adriatic coast islands (30,31), or isolated Slavic populations like the Lužice Sorbs (32), than the surrounding Central European populations of the Czech Republic, Slovak Republic, or Poland. Zalán et al (33) have also reported such low values of Y-chromosomal diversity in Hungarian Vlax Roma (not related to the Valachs, despite the similar name). A common feature of all these populations is geographical and/or cultural isolation, which in the case of the Moravian Valachs lasted at least until the beginning of the 20th century. The isolation of the Valachs from the surrounding populations was already documented by F_ST_ pairwise distances. Non-significant pairwise F_ST_ genetic distances based on 12 Y-STRs were shown between the regions surrounding the Valach country (Moravia-Silesia and Zlin regions, data by Zastera et al) and CZE, but highly significant between the Valach samples and both CZE and Zastera’s data (*P* < 0.010; 10000 permutations). Thus the Valachs are isolated not only from the general Czech population, but also from the non-Valach Czech population from the regions neighboring the Valach region.

Y-STR analysis of the VALACH population sample revealed a previously unnoted modal haplotype within the haplogroup R1a (VMH). The reason for comparing the VMH with the European metapopopulation of Y-chromosome haplotype reference database was to test whether the VMH was specific just for the typical Moravian Valach region or can be found in populations where we expect the occurrence of Valach individuals. The majority of close and neighbor matches were from the populations and geographic locations where the migration of the Romanian or Moravian Valachs was recorded. The matches in non-Slavic regions (Argentina, Spain, Italy, Germany) are in concordance with the emigration waves of the Valachs in the 19th and 20th century.

Forensic analysis of Y-chromosome loci requires the highest possible haplotype diversity. Some studies have shown (34) that commercially available kits like PowerPlex Y or Yfiler cannot provide a sufficient discrimination power to discern the haplotypes inside broad families, and a definition of a region-specific set of Y-STR loci is a must. The results of haplotype diversity of the population samples tested within this study (VALACH, VLIN, and CZE) revealed that the sampling in one close geographic region or inside a group of people that recognize themselves as members of a certain “clan” brings lower diversity. However, this phenomenon enables us to find a founding haplotype for that group. The modal haplotype within VALACH population sample belonging to the haplogroup R1a can be of high forensic importance as it defines a relatively large group of individuals that can be identified through Y-chromosome STR analysis of modality. Defining such modal haplotype is only possible in the case of detailed knowledge of the genetic (sub)structure of the population in question.

Besides the genetic or forensic aspects, the ethical aspects of data gathering and presentation should be predominant, especially if we want to investigate not-so-distant paternal lineages. Moreover, genealogical, linguistic, and historical information is also of foremost interest to the researcher. The optimal number of Y-chromosomal STR polymorphic loci to be used in such a study differs vastly according to the objectives. We confirmed that 17 loci of Yfiler kit could be insufficient for forensic application if we were to analyze a cluster of related paternal lineages. On the other hand, for population genetic applications, a set of a few precisely defined core Y-STR could very well describe the population and could be used for interpopulation comparison. Properties of the sample set under examination are also strongly influenced by the sampling procedure, which is not always known or properly administered. We demonstrated this sampling effect on the differences between our Moravian Valach sample sets (VALACH vs VLIN).

Our analyses confirmed that the Moravian Valachs represent a unique population data set from the Czech Republic and the whole region of Central Europe due to their ethnographic coherence and isolation that is clearly detectable in their Y-chromosomal diversity. The VMH can be used for further studies on Valach migration or in the evaluation of forensic analysis results.

## 

**Table:** 

	SLO	SAX	POLC	POSE	HUN	VALACH	VLIN	CZE
**SLO**	-	0.03393	0.01043	-0.00571	**0.05227**	0.01966	0.04191	-0.00406
**SAX**	**0.02052**	-	**0.05743**	0.02967	-0.00808	0.00198	0.00897	0.03808
**POLC**	0.00188	**0.03079**	-	-0.00474	**0.07888**	0.03122	**0.10654**	0.00163
**POSE**	0.00532	**0.03252**	-0.00043	-	**0.04802**	0.01960	-0.00224	-0.00355
**HUN**	**0.02450**	0.00532	**0.03532**	**0.04190**	-	0.00270	0.02325	**0.06064**
**VALACH**	**0.08979**	**0.05805**	**0.10999**	**0.10114**	**0.08175**	-	0.00623	0.03159
**VLIN**	**0.02830**	**0.06106**	**0.04693**	**0.05861**	**0.06207**	**0.14301**	-	-0.00510
**CZE**	**0.01108**	-0.00114	**0.01783**	**0.02046**	0.00482	**0.06421**	**0.04759**	-

*Statistically significant pairwise distances are printed in bold (*P* < 0.01; 10 000 permutations).
